# Current Understanding of Aortic Dissection

**DOI:** 10.3390/life12101606

**Published:** 2022-10-14

**Authors:** Xun Yuan, Andreas Mitsis, Christoph A. Nienaber

**Affiliations:** 1 Cardiology and Aortic Centre, Royal Brompton and Harefield Hospitals, Guy’s and St Thomas’ NHS Foundation Trust, London SW3 6NP, UK; 2 National Heart and Lung Institute, Faculty of Medicine, Imperial College London, London SW7 2BX, UK; 3 Cardiology Department, Nicosia General Hospital, Strovolos 2029, Cyprus

**Keywords:** aortic dissection, diagnosis, TEVAR, surgery

## Abstract

The aorta is the largest artery in the body, delivering oxygenated blood from the left ventricle to all organs. Dissection of the aorta is a lethal condition caused by a tear in the intimal layer of the aorta, followed by blood loss within the aortic wall and separation of the layers to full dissection. The aorta can be affected by a wide range of causes including acute conditions such as trauma and mechanical damage; and genetic conditions such as arterial hypertension, dyslipidaemia, and connective tissue disorders; all increasing the risk of dissection. Both rapid diagnostic recognition and advanced multidisciplinary treatment are critical in managing aortic dissection patients. The treatment depends on the severity and location of the dissection. Open surgical repair is the gold standard of treatment for dissections located to the proximal part of the aorta and the arch, while endovascular interventions are recommended for most distal or type B aortic dissections. In this review article, we examine the epidemiology, pathophysiology, contemporary diagnoses, and management of aortic dissection.

## 1. Introduction

The aorta is the body’s largest blood vessel, and its primary function is to carry oxygen and blood from the left ventricle to other organs of the body. Anatomically, the aorta is divided into four parts: the ascending aorta (which starts from the heart and from which coronary arteries arise), the aortic arch (which bends over the heart and turns towards the posterior thoracic wall), the descending thoracic aorta (which extends through the posterior thoracic cavity in vicinity of the spine) and finally, the abdominal aorta (which runs below the diaphragm) [1,2]. Clinically, aortic dissection is classified by the location of the dissection and/or origin of the intimal tear and the expanse of the dissection. Stanford type A (or DeBakey type I and II) involves the ascending aorta, while Stanford type B (or DeBakey IIIa and IIIb) involves the descending thoracic and abdominal aorta [3,4].

**Figure 1:**
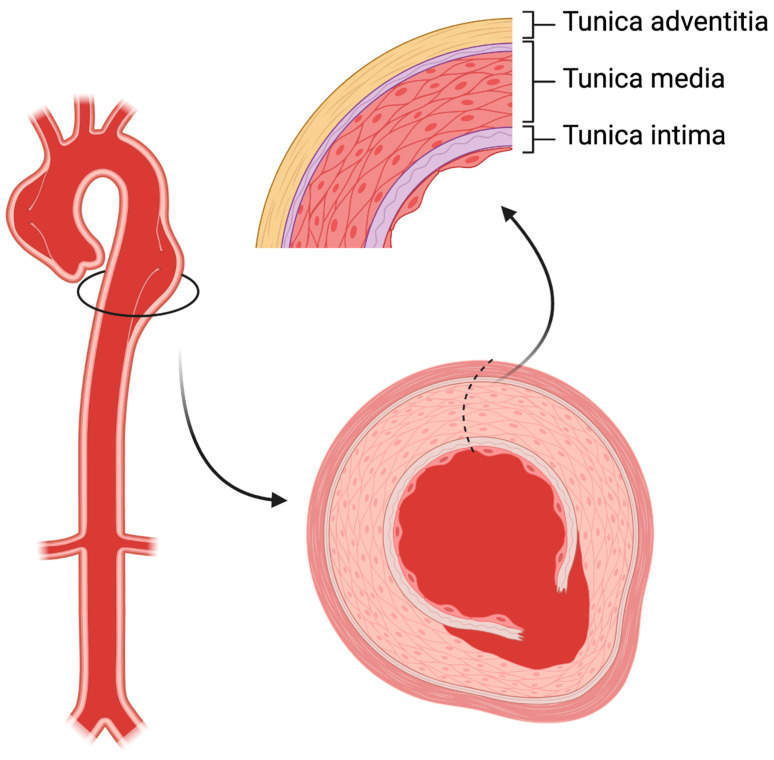
The structure of aortic wall. In aortic dissection, a tear in the intima layer results in blood entry to the media layer, developing in an intimal flap and dividing the original vessel into true and false lumen.

The aortic wall comprises three layers: the thin tunica intima which faces the bloodstream, the thick musculo-elastic tunica media, and the outer fibrous tunica adventitia (Figure 1). In acute aortic dissection, a tear in the intima layer results in blood entry to the media layer, developing in an intimal flap and dividing the original vessel into true and false lumen. A broad range of diseases can affect the aorta, including acute (such as trauma and hypertensive emergencies) or chronic conditions (such as chronic arterial hypertension, connective tissue disorders, inflammatory vasculitis and atherosclerosis). Aortic dissection is considered a complex vascular scenario that benefits in terms of prognosis from a rapid diagnosis and advanced teamwork including cardiac surgeons, cardiologists, vascular interventionists, and radiologists. This review will focus on current understanding the incidence, pathophysiology, and management of aortic dissection. We will describe observations of traditional and future strategies to improve prognosis and the long-term outcomes of this difficult condition.

## 2. Epidemiology

Population-based studies in the US and Europe indicate an incidence of 2.6 to 3.5 cases per 100,000 person-years [5,6]. Another study from the UK observed 92,728 patients over 10 years, and reported a higher incidence (6 per 100,000 person-years) of acute aortic dissection [7], which is similar to data from Sweden with an incidence of 7.2 per 100,000 person-years [8]. The real-world incidence may be underestimated due to omission of pre-admission deaths in all studies. Interestingly, the demographic forecasts from the UK Office for National Statistics predicts that the incidence of aortic dissection will rise from 3892 in 2010 to 6893 in 2050 in both men and women, with the majority occurring in individuals over 75 [7,9]. The number of cases of acute aortic dissection seems to be rising in Western countries, possibly related to increased awareness of the disease, as well as access to the use of advanced imaging techniques (particularly CT) in emergency departments [10,11,12].

The frequency of acute aortic dissection is notably higher in males, in particular those who are older [8,13]. Women are usually older than men at presentation, with a mean age of 72 years vs. 64 years in men [14,15]. Despite the higher rates of aortic dissection being in men, it is women who have a higher mortality rate. Data from the International Registry of Aortic Dissection (IRAD) indicates that women with aortic dissection typically present at hospital later than men with worse clinical status (coma and tamponade). The data also show they have different types of symptoms and show less typical clinical presentation [16]. These data may partly be explained in a different analysis; after age-adjustment, women have higher rates of mortality then men and more often suffer pre-hospital death [8,16].

## 3. Classification

**Figure 2:**
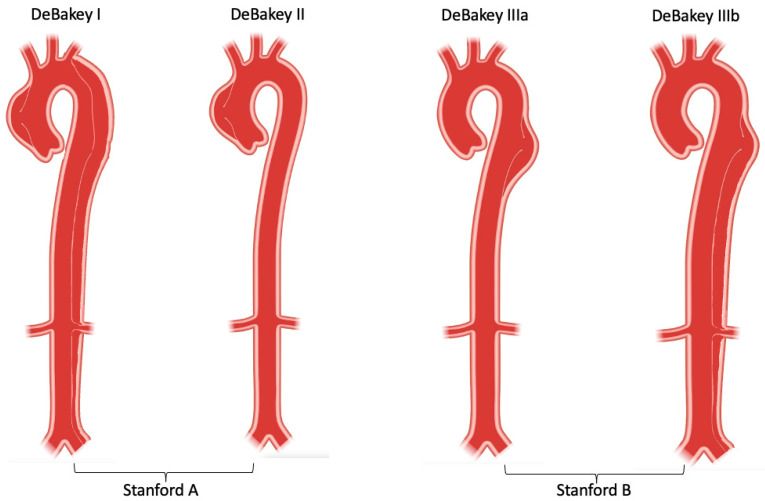
DeBakey and Stanford aortic dissection classification.

Aortic dissection can be categorized in different ways in terms of anatomy and symptom onset. The two most frequent anatomic classification systems are: DeBakey classification, which is based on the site of origin of the intimal tear [4]; and Stanford classification, which specifies the involvement, or lack thereof, of the ascending aorta [3]. DeBakey classification precisely describes the site of the dissected segment or diseased lesion: Type I dissections usually originate from the ascending aorta and have the most extensive involvement including the ascending aorta, aortic arch, descending aorta and further; Type II dissections originate from and are only limited to the ascending aorta; Type III dissections originate from the descending aorta after the left subclavian artery orifice, and affects the descending aorta (Type IIIa) and/or distal abdominal aorta (Type IIIb) [4]. Stanford classification helps indicate different management for dissection cases in clinical practise: Type A dissections involve the ascending aorta (DeBakey type I and II), usually requiring swift surgery; while Type B dissections only involve the descending aorta (DeBakey type III) and can be managed endovascularly or medically [3,17] (Figure 2). Recently, a unique group, so-called non-A non-B, which does not completely fit DeBakey and Standford classification has been identified. These patients develop a dissection originating from the aortic arch, or the descending aorta and retrogradely involve the arch, without involving the ascending aorta. Several studies suggest that arch dissections could lead to organ malperfusion and aortic rupture, which require prompt intervention and careful management [18,19]. A new classification system, which includes the type of dissection (adapting Stanford system A, B or non-A non-B), the location of the primary entry tear, and the presence of malperfusion (TEM) has been introduced, but is yet awaiting community acceptance [19].

Clinically, type B aortic dissection are considered complicated or uncomplicated depending on high-risk features. Almost a quarter of type B patients present with some high-risk features such as signs of imminent rupture, evidence of organ or limb ischemia, refractory hypertension, hypotension (systolic blood pressure < 90 mmHg), and cardiogenic shock, which require early intervention to treat malperfusion or ischemia so as to avoid death [20]. Uncomplicated type B dissection is traditionally treated medically with close, regular surveillance. However, more and more studies show that morbidity and mortality could be as high as 30% and 42%, respectively, after 5 years of conservative management in uncomplicated type B dissection [21,22,23]. The INSTEAD-XL trial demonstrated significant survival benefit in the TEVAR group compared with medical only [24]. The ADSORB trail showed the feasibility and safety of TEVAR treatment for uncomplicated type B dissections in acute phase [25]. An imaging analysis of the ADSORB trial helped identify uncomplicated type B dissection patients who are at high-risk of developing aortic events, and also revealed better false lumen thrombosis in the TEVAR group than medical only (90.3% vs. 31.0%) [26]. As the vascular community understand the natural progress of type B dissection better, the term “uncomplicated” is under debate, and more point-based, outcome-oriented category systems are yet to be introduced [27,28,29].

Based on the period of onset of symptoms and diagnosis, the binary classification of the acute period (<2 weeks) and chronic period (>2 weeks) has been in use for decades [30]. As diagnostic technology and management improved, a new temporal classification based on patient survival rates from IRAD was introduced. According to the time of symptom onset, aortic dissections can be classified into hyperacute (<24 h), acute (2–7 days), sub-acute (8–30 days), and chronic (>30 days) phases [31]. Following symptom onset, survival continues to decrease considerably up to 30 days after presentation and falls into what has been traditionally considered the “chronic” phase of aortic dissection. This finding appears to be present in both Type A and Type B aortic dissections irrespective of the treatment strategy. Of note, this time approximately relates with the average length of hospital stay in these patients, meaning that close to discharge, even relatively “stable” patients have significant mortality risk. For these patients, continued surveillance is paramount. Attention should be given to aggressive use of both medical and nonmedical treatment modalities beyond the hyperacute and acute time periods, and into the traditional chronic phase. To sum up, stratifying aortic dissection into these four chronological groups offers a more sophisticated assessment of survival as it evolves over the first 60 days following symptom onset. This classification indicates patient survival rate and outcome, ultimately helping individual decision-making for early and late treatment decisions regarding the management plan of acute aortic dissection [31].

## 4. Pathophysiology

The fundamental physiopathological rule underlying aortic dissection is an elevation in blood pressure leading to the separation of the layers of the media. The latter results in the formation of a false lumen within the aortic wall. The two main predisposing factors related to its development are a history of structural weakness of the aortic wall, and increased wall tension on the aortic wall.

### 4.1. Genetics

A wide spectrum of connective tissue diseases such as Marfan syndrome, Loeys–Dietz syndrome (LDS), and type IV Ehlers–Danlos Syndrome (EDS) are known as predisposing factors for both aortic aneurysms and dissections [2,32].

Marfan syndrome is an autosomal dominant genetic condition caused by mutations in FBN1 [33,34] or FBN2 genes [35,36]. Marfan syndrome was reported in 4–5% of aortic dissection patients [11,37]. FBN1 and FBN2 genes encode fibrillin-1 and fibrillin-2, components of elastin-associated microfibrils that can mainly be found in the tunica media. Marfan syndrome is characterised by a tendency to develop predominantly aortic diseases (aneurysms and dissections) and special skeletal and ocular features, which are mainly caused by FBN1 gene mutations [33]. Patients with Marfan syndrome are usually younger compared to non-Marfan patients (aged 38.2 ± 13.2 years versus 63.0 ± 14.0 years; *p* < 0.001) at time of dissection, and present with fewer comorbid conditions (atherosclerosis and hypertension) [37].

LDS has many similarities to Marfan syndrome, as both are inclined to develop dissections and aneurysms of other arteries as well as the aorta [36]. It is a relatively new autosomal dominant disorder that portrays the consequences associated with mutations in transforming growth factor beta receptor genes 1 and 2 (TGFBR1/TGFBR2) [38,39]. Interestingly, almost 98% of patients with LDS were found to have aortic root aneurysms, which makes them highly susceptible to the development of an aortic dissection [40,41].

Finally, EDS is characterised by a tendency for vulnerability to and rupture of arteries, intestines, and uterus [42]. It is an autosomal dominant disorder that impacts the extracellular matrix of the cardia and vasculature through defective type III procollagen gene (COL3A1), which is responsible for almost all cases. Among the different types of the syndrome, type IV EDS (or vascular EDS) has the worst prognosis [43].

### 4.2. Chronic Inflammation

Apart from genetic effect, a chronic inflammatory status also contributes to simulating the process of medial degeneration by immunologic effector cells [44].

Central to the pathophysiology of dissection are pro-inflammatory cells and express cytokines that directly trigger aortic wall degradation through smooth muscle cell apoptosis and extracellular matrix proteolysis [45,46,47,48].

When aortic wall cells are placed under physiological pressure observed in aortic dissection, apoptotic and inflammatory signalling pathways are activated [49]. In addition, the pro-inflammatory condition presenting with apoptotic smooth muscle cells urges lymphocytes, macrophages, and other inflammatory cells to infiltrate the tunica media from the adventitia [50]. This leads to the deterioration of the wall structure and aneurysmatic degeneration of the aorta, which are prone to dissection and rupture. Moreover, apoptotic smooth muscle cells can recruit such inflammatory cells and secrete pro-inflammatory proteolytic enzymes such as elastase and collagenase, resulting in degradation of the extracellular matrix [44,48,51].

### 4.3. Arterial Hypertension

Several conditions are known to be linked to elevated aortic wall stress. Among them, uncontrolled or untreated arterial hypertension is without doubt the most common treatable pre-morbid risk factor [10]. Almost 75–80% of patients with aortic dissection present with a history of arterial hypertension. Of note, this is more frequent in type B aortic dissection than in type A aortic dissection (80.9% versus 74.4%; *p* < 0.001) [10,52]. In addition, arterial hypertension has been linked to atherosclerotic degeneration of the aorta, leading to fragility of the aortic wall, which includes fibrosis, intimal thickening, calcification, extracellular fatty acid deposition, and extracellular matrix degradation that compromise the elastic properties of the wall [53]. Hypertension may also contribute to matrix metalloproteinases (MMPs) and the production of pro-inflammatory cytokines which lead to excessive extracellular matrix degradation [53].

A recent meta-analysis comprising over 1 million individuals from both Western and Asian gene pools reconfirmed that hypertension and both elevated systolic and diastolic blood pressure are associated with an elevated risk of aortic dissection. Even with blood pressure kept at the upper range of normal, has been defectively linked to being riskier than blood pressure at the lower end of normal [54].

### 4.4. Bicuspid Aortic Valve

Bicuspid aortic valve (BAV) disease is the most frequent congenital cardiac disorder, with prevalence at birth ranging between 1–2% (male-to-female ratio 2:1 to 4:1) [32,40]. BAV is the most common congenital heart abnormality, resulting in the majority of morbidity and mortality caused by congenital heart defects [55]. Especially, BAV is a well-known risk factor for ascending aortic and root dilatation, aneurysmal degeneration formation, and aortic dissection or rupture [32,56]. Interestingly, the most common phenotype (in almost 60–70% of BAV patients) is an aneurysm involving the tubular ascending aorta, with the fastest growing rate in adults (≈0.4–0.6 mm/y). This phenotype is independent of valve pathophysiology and function. However, the entire ascending aorta may be affected, including sinuses of Valsalva and tubular aorta with sinotubular junction effacement. Finally, there is another phenotype that is less common (≈25% of BAV patients) and associated with type 1 (right-left cusp fusion) BAV morphology and male gender. This phenotype, since it affects the root, has been related with faster tubular-ascending aorta dilatation, and aortic regurgitation [57]. Compared to the normal population, BAV individuals tend to have much higher aortic dissection incidence (8.4 of relative risk) [58]. In particular, the root phenotype with aortic regurgitation has recently been associated with a higher risk of aortic dissection. Data from IRAD showed that among 3393 patients with aortic dissection, 113 (3.3%) had BAV, containing 93 patients (82.3%) with type A aortic dissection and 20 patients (17.7%) with type B aortic dissection [59].

## 5. Diagnosis

One of the most important diagnostic challenges of patients with acute aortic dissection is the low prevalence, followed by often nonspecific clinical presentation, and the lack of specific biomarkers [60]. Thus, there is an elevated risk of misdiagnosis with associated increased risk of serious consequences. To improve those diagnostic delays, acute aortic dissection training modules and continuing education programs of all the stakeholders including physicians at emergency departments, radiologists, and cardiovascular specialists are crucial. Thus, the use of a standard diagnostic algorithm for acute aortic syndrome is paramount [61].

Since the poorer outcomes such as morbidity and mortality are heavily associated with treatment delay, early diagnosis is essential in initiating proper management. A comprehensive analysis of predisposition factors of aortic dissection including imaging, biomarkers, and genetic defects is critical for any given patient to establish diagnosis and select an appropriate diagnostic intervention. It is also important to determine high-risk features such as signs of impending rupture, the extent of dissection, branch vessel involvement, and any ischaemia or malperfusion in end-organ and the extremities, because they contribute to management decision-making.

### 5.1. Clinical Presentation and Complications

The presentation of aortic dissection can vary and depends on age, comorbidities and complexities. The classical presentation is characterized by an acute onset and severe, tearing pain in the chest or back [32]. The majority type A dissection patients experience a sudden onset of chest pain with characteristics of sharp or stabbing pain, while type B dissection patients present with localized back pain or pain migrating to the abdomen, which suggests the dissection extends to the distal segment of the aorta. Acute onset chest pain with radiation is a significant positive predictor associated with acute aortic syndrome (odd ratio = 11.7, sensitivity 82.9%, specificity 70.7%), which should alert physicians to the suspicion of aortic dissection after ruling out myocardial infarction [62]. Nevertheless, 6.4% dissection patients were reported painless [63], 62% of which were male with a mean age of 64.8 [64]. Among those painless patients, left-sided neurological deficits (21%) were found to be the most frequent presentation symptom, followed by dyspnoea and bilateral neurological deficits of the lower extremities, in 18% and 15%, respectively [64]. Pulse deficits, reported in up to 30% of patients according to IRAD [63], are highly indicative of aortic dissection with a positive likelihood ratio of 5.7 [65]. Subsequently, other ischemia and malperfusion such as cerebral, visceral, end-organs, spinal cord, and extremities should be considered and ruled out by physical and neurological examinations.

Both hypotension (reported as up to 30% in IRAD) [63] and syncope (occurs in 15% of dissection patients, specially in type A dissection) [32] are most worrying symptoms. Patients presenting with syncope and/or hypotension usually indicate poor outcomes (increased in-hospital mortality) due to underlying conditions such as cardiac tamponade and aortic rupture, which require immediate intervention [66,67].

### 5.2. Imaging Modalities

Chest X-ray and echocardiography have markedly low diagnostic accuracy and can be completely normal in a large number of patients with aortic dissection (28% and 39%, respectively) [10,11]. Computed tomography angiography (CTA) has higher diagnostic accuracy, is broadly available in emergency departments, and is easily and swiftly accessible [68]. Importantly, the use of CTA as the initial diagnostic imaging modality in type A dissection increased from 46% to 73% (*p* < 0.001) between 1996 and 2013, and was used in 80–85% of type B aortic dissections [10,11]. Therefore, CTA, best with electrocardiogram (ECG)-gated image acquisition, is widely accepted as the first-line diagnostic imaging modality in an acute setting and should be used at low threshold. Transoesophageal echocardiography and magnetic resonance imaging (MRI) or invasive aortography are less often used modalities when establishing an acute diagnosis [68,69,70]. MRI and magnetic resonance angiography (MRA) are recommended over CTA for subacute and chronic aortic dissection patients due to less exposure to ionizing irradiation, especially in young patients. Like CTA, MRA has also been found to have close to 100% sensitivity and specificity. However, since it is more time-consuming, it is usually used for following up the condition. Particularly, contrast-enhanced MRA is able to obtain sufficient signals in bent and turbulent flow regions. Furthermore, cine MRI may be used to evaluate the entry in aortic dissection. In comparison to other imaging modalities, MRI/MRA can provide additional functional information such as hemodynamics and biomechanics on top of anatomy, which could contribute to stratifying individual risk and personalised long-term management for dissection patients [71,72,73].

### 5.3. Laboratory Biomarkers

Biomarkers have a role in both diagnostic and prognostic purposes, not only functioning as early risk predictors of aortic dissection, but also showing their potential as new tools to detect aortic dissection [74,75,76]. When aortic dissection occurs, endothelium releases medial smooth muscle cellular components into the circulation; some smooth muscle cell markers such as smooth muscle myosin heavy chain and calponin can be detected from the peripheral serum [77,78]. Soluble elastin fragments and MMPs, which are extracellular matrix proteins can also be detected during aortic injury [79,80,81,82]. D-dimers is a thrombosis (fibrinolysis) marker widely used in clinical setting; its potential for reflecting dynamic coagulopathic states induced by aortic wall injury makes it a candidate for aortic dissection [83]. However, the diagnostic role of D-dimers is limited due to a pooled sensitivity (94% for dissection) and varying specificity (40–100%) [83,84]. C-reactive protein, an inflammatory biomarker, increased after dissection, which potentially reflects the extent of damage to the aorta and ongoing systemic inflammation [85,86]. Interleukin 6 originating from the liver after cytokine stimulation, was found to be associated with the severity of dissection and the time after dissection onset [87,88,89]. Newly found biomarker ST2 (suppression of tumorgenicity 2) has shown its potential role in diagnosing aortic dissection and is currently under further investigation [75,90].

## 6. Management

In the hyperacute phase (within the first 24 h), patients with aortic dissection require intensive monitoring in a cardiovascular care unit in preparation for swift surgery in case of proximal dissection. The most important aspects of treatment are hypertension control, pulse rate control, and pain relief [32,40]. In order to achieve a rapid lower blood and pulse control, the use of intravenous beta-blockers simultaneously combined with antihypertensive therapy is paramount. Sedatives should always be considered, and serum lactate levels should be monitored [1,10,74].

### 6.1. Type A Aortic Dissection

Generally, type A aortic dissection is considered a life-threatening condition, which requires emergency open surgical repair. The patient’s condition and the segment of the aorta play a crucial role in the operative technique used. The principal objective is to avert death and aortic rupture and to correct aortic regurgitation (if present), as well as any organ malperfusion. This is most commonly achieved by replacement of the ascending aorta and hemiarch together with aortic valve resuspension.

**Figure 3:**
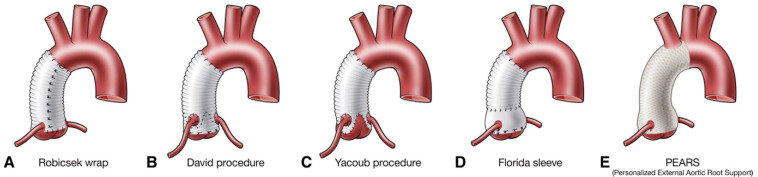
Valve-sparing aortic root surgeries. Figure reproduced from Buratto E., Konstantinov I.E. Valve-sparing aortic root surgery in children and adults with congenital heart disease. *J. Thorac. Cardiovasc. Surg.* **2021**, *162*, 955–962.

When concomitant connective tissue disorders are present, aortic root replacement with a biological or mechanical valve graft for the replacement of the aortic valve, aortic root, and ascending aorta is advised. In this scenario, the coronary arteries are reimplanted into the composite graft. This is achieved using a modified version of the Bentall–de Bono technique, which is currently the standard treatment option in type A aortic dissection involving the aortic root [91]. However, there are cases where the sinuses of Valsalva are spared from the disease, in which case an interposition graft is usually sufficient; consecutively, the aortic valve may be replaced or reinstated. In selected patients with favourable valve anatomy, valve-sparing aortic root operations are an alternative to a composite valve tube when in the hands of experienced surgeons (Figure 3).

The standard approach for the management of a type A aortic dissection continues to be hemiarch replacement with open distal anastomosis (proximal arch repair without involving the arch vessels). It is thought to be safer than total arch replacement due to being less invasive, and its simplicity and reproducibility make it the choice of operation for suitable patients. In an effort to reduce the instance of late reinterventions, several groups advise total replacement the aortic arch in patients with presence of dilated arch, extensive arch tears, or branch vessel dissection [91,92]. Lastly, a novel method, called the “frozen elephant trunk” technique, using a hybrid prosthesis, permits the sealing of re-entry tears in the descending aorta, forwards blood flow to the thoracoabdominal aorta true lumen, and permits the repair of the aortic arch and proximal descending aorta, thereby promoting false lumen thrombosis [93].

### 6.2. Type B Aortic Dissection

Traditionally, in cases of uncomplicated type B aortic dissection, optimal medical therapy has been used [32]. European guidelines advise that in complicated type B aortic dissection, the recommended treatment is thoracic endovascular aortic repair (TEVAR) [32,94]. Of note, cases of uncomplicated type B aortic dissection may also benefit from TEVAR in order to prevent late aortic complications [24,94]. However, its prognostic benefit has yet to be proven. The suitable timing for TEVAR in uncomplicated type B aortic dissection is still under continuous debate. When preformed in later phases it has shown adequate aortic remodelling with a low periprocedural complication rate [17,24]. Furthermore, the use of TEVAR still remains uncertain in patients with connective tissue disorders [37,95]. However, in an emergency setting, TEVAR may be considered in patients with connective tissue diseases [96,97]. The group of patients with retrograde extension from the entry tear in the descending aorta to the ascending aorta with a completely or partially thrombosed false lumen in the ascending aorta can initially be managed medically [98]. However, many patients with arch dissection have a complicated disease course [99]. Positive short-term results have been found in clinical scenarios using hybrid repair, open surgery (frozen elephant trunk), or endovascular techniques [100,101].

### 6.3. New Potential Approaches

#### 6.3.1. TEVAR in Type A Aortic Dissection

Although open surgery repair remains the gold standard for the management of acute type A aortic dissection, there are elderly patients or individuals with many comorbidities, in which surgical treatment may be considered extremely high risk or futile. Endovascular treatment has been applied to a limited number of patients. Data from this approach have been limited to case reports and small series [102,103]. Technical challenges are presented by the dynamic motion of the ascending aorta and the proximity of anatomic structures to intended landing zones (aortic valve, coronary arteries, and supra-aortic vessels). For this reason, specially designed endografts to address these issues are still not available, resulting in the application of endovascular therapies to ascending aorta being currently limited [104]. While TEVAR has substituted open aortic repair for a suitable lesion in distal aortic dissection, some selected patients with type A aortic dissection at high surgical risk may be candidates [105].

#### 6.3.2. Personalised External Aortic Root Support (PEARS)

Personalised External Aortic Root Support (PEARS) is a prophylactic surgery using a customised soft macroporous mesh sleeve to support and secure the aorta in patients with a dilated aortic root and ascending aorta. 16 years of surveillance and the follow-up of almost 400 cases showed extremely positive and reassuring results in respect to halting dilation and thereby minimising dissection risk in the future [106].

#### 6.3.3. False Lumen Interventions to Promote Remodelling and Thrombosis (FLIRT)

To prevent further cases of false lumen dilatation and the potential for ruptures in the long term, the therapeutic provocation of complete thrombosis, by occlusion of entry or re-entry tears, is a promising approach. The traditional endovascular method is the occlusion of connections between true and false lumen by stent–graft coverage, possibly followed by the use of custom-made fenestrated and branched endografts at the level of abdominal re-entries. A new concept of the endovascular approach with vascular occluder devices and coils has shown early feasibility and benefits in selected type A aortic dissection and post-TEVAR residual dissection patients [107,108].

### 6.4. Follow-Up and Surveillance

Since the entire aorta and its branches could be affected in a various ways such as dissection, aneurysmatic degeneration, and rupture, aortic dissection should be treated as a systemic condition. Literature reported a 10-year survival rate of acute aortic dissection ranges from 30–60% [9,11,14,109]. Therefore, delicate follow-up and life-long surveillance play a critical role in patients’ long-term outcomes.

Optimised medical management remains the foundation of aortic dissection treatment, even after successful surgical or endovascular treatment, adequate medication including beta-blockers, angiotensin-II-receptor antagonists, and statins is essential to maintaining controlled blood pressure and inflammation [32]. All aortic patients should be followed up clinically with repeating images on a regular basis to minimise risk factors and recognise progression of aortic events early. Especially for those who have residual dissection after treatment or are initially managed conservatively.

Imaging weighs a significantly in surveillant protocol. Chronic dissection patients could gain prognostic benefit from the timely detection of asymptomatic progression, which allows instant surgical repair or endovascular intervention to avoid rupture [1]. Post-surgery or intervention patients should be focused on procedural-related complications such as suture aneurysm and endoleak in the first 2 years after procedure, and then maintain less strict imaging intervals [32]. ECG-gated CTA over MRI/MRA is the modality of choice, with better visualisation of endografts and less artefacts [71,110]. It is important to understand that possible expansion of the false lumen, in the case of dissection or retrograde progression of a chronic distal dissection, is often subclinically silent, but could be harmful if not managed properly. On the other hand, proof of remodelling of dissected aorta after endovascular or open repair may indicate an excellent long-term prognosis [24].

Aortic dissection patients with genetic defects require more extensive attention and personalised solutions including lifestyle measures, medication, family planning and pre-emptive surgery. The genetic counsellor usually sees patients/families with a confirmed hereditary diagnosis and indication for pre-symptomatic genetic counselling and cascade testing, or if pre-conceptual counselling is required to go through the options available prior to starting a family. Moreover, the genetic counsellor plays an important role in liaising with family member if necessary to complete pedigrees and/or organize segregation analysis involving multiple family members (who need to be tested to find out whether a genetic variant is indeed disease-causing or not).

Aortic dissection patients usually suffer from both physical and psychological issues for a long time, even after satisfactory treatment [111]. This might result from the stress incited by the morbid nature of disease, and the trauma of dramatic experience [112,113]. Patient-centred rehabilitation and continual mental support are recommended as part of a multi-disciplinary team approach to tackling the problem.

## 7. Outlook

Significant progress has been made over the last two decades in the identification and treatment of patients with aortic dissection. However, important gaps continue to remain in the management of acute aortic pathologies. We anticipate new emerging imaging tools and simulation algorithms to further improve our knowledge of the aortic wall structure, its functional properties and reactions to wall stress. Specific aortic dissection biomarkers are critically needed to detect the disease at its earliest stage. Reference to aortic centres for aortic surgery and working with an aorta flowchart should be encouraged. Further studies are needed to advance the progress and management of patients with the less common forms of aortic dissection. Either compulsory registries or prospective multicentre clinical trials are required to test the efficacy of preventive interventions in the setting of aortic conditions.

## Data Availability

Not applicable.
